# Impact of Strain Competition on Bacterial Resistance in Immunocompromised Populations

**DOI:** 10.3390/antibiotics9030114

**Published:** 2020-03-07

**Authors:** Ashley A. DeNegre, Kellen Myers, Nina H. Fefferman

**Affiliations:** 1 Department of Ecology, Evolution and Natural Resources, Rutgers University, New Brunswick, NJ 08901, USA; ashley.denegre@gmail.com; 2 The Command, Control and Interoperability Center for Advanced Data Analysis (CCICADA), Rutgers University, New Brunswick, NJ 08901, USA; 3 Department of Ecology & Evolutionary Biology, University of Tennessee, Knoxville, TN 37996, USA; kmyers@tusculum.edu; 4 Department of Mathematics, University of Tennessee, Knoxville, TN 37996, USA; 5 National Institute for Mathematical and Biological Synthesis (NIMBioS), University of Tennessee, Knoxville, TN 37996, USA; 6 Department of Mathematics, Tusculum University, Greeneville, TN 37745, USA

**Keywords:** mathematical models, evolutionary epidemiology, resistant opportunistic infections, AIDS-related opportunistic infections, global public health, emerging drug resistance, chemoprophylaxis

## Abstract

Despite the risk of emerging drug resistance that occurs with the frequent use of antimicrobial agents, targeted and prophylactic antibiotics have been considered crucial to opportunistic infection management among the HIV/AIDS-immunocompromised. As we recently demonstrated, the disrupted selective pressures that occur in AIDS-prevalent host populations increase the probability of novel emergence. This effect is concerning, given that bacterial strains unresponsive to first-line antibiotics can be particularly dangerous to hosts whose immune response is insufficient to fight infection in the absence of antibiotic support. While greater host susceptibility within a highly immunocompromised population may offer a fitness advantage to drug-resistant bacterial strains, this advantage could be mitigated by increased morbidity and mortality among the AIDS-immunocompromised. Using a Susceptible-Exposed-Infectious-Recovered (SEIR) epidemiological model parameterized to reflect conditions in an AIDS-prevalent host population, we examine the evolutionary relationship between drug-sensitive and -resistant strains of *Mycobacterium tuberculosis*. We explore this relationship when the fitness of the resistant strain is varied relative to that of the sensitive strain to investigate the likely long-term multi-strain dynamics of the AIDS-mediated increased emergence of drug resistance.

## 1. Introduction

Among HIV/AIDS immunocompromised patients, the frequent use of antibiotics is essential in the prevention and/or treatment of many opportunistic bacterial pathogens [1]. Yet, it is well-known that with increased antibiotic use comes the increased likelihood of selection favoring the emergence of antibiotic resistance [2,3,4]. Though this risk is markedly greater when antibiotics are used incorrectly—as in the cases of over-prescription or patient non-adherence to dosing instructions—resistance may still arise out of appropriate antibiotic use, especially in the case of chronic, prolonged illness [5,6,7].

We recently demonstrated that the disrupted selective pressures associated with an AIDS-prevalent host pool can drastically increase the probability of the emergence of antibiotic resistance [8]. Emerging resistance has the potential to be particularly devastating in HIV/AIDS-prevalent regions due to widespread host immunoincompetence. Pathogen nonresponse to one or more first-line antibiotics promotes the maintenance of resistant strains within a host population whose collective immunosuppression offers little to no defense against the spread of infection. Although morbidity and mortality associated with resistant infection may be higher among the AIDS-immunocompromised, the emergence of resistance also poses a risk to immunocompetent susceptibles. Even in immunocompetent hosts, immune activation can sometimes be insufficient to effectively fight infection without the support of antibiotics. However, when immune response alone fails to adequately clear an infection caused by an antibiotic-resistant pathogen strain, treatment options are critically limited [9].

In our previous works [8,10], we made the simplifying assumption that antibiotic-resistant infections, while originating via the emergence of drug-resistant mutations, increase in prevalence solely due to selective pressures within the host population. However, these same selective advantages can lead to resistant strain dominance, which in turn leads to a greater percentage of infectives harboring and transmitting pathogens that are nonresponsive to antibiotics [11]. Using *Mycobacterium tuberculosis* as a model pathogen, we now examine the impact of the emergence and maintenance of resistance via bacterial strain circulation and the potential for strain replacement.

We have chosen to focus on the developing world because, within resource-limited settings, poor sanitation and infection management enhances the burden of infectious disease, and economic constraints can hinder access to effective antibiotics [12]. Moreover, factors such as a limited understanding of HIV transmission, high-risk sexual behavior (sometimes in conjunction with intravenous drug use), and inconsistent access to highly active antiretroviral therapy (HAART) once seropositive, currently place developing nations in danger of increasing HIV/AIDS prevalence [13,14]. The combination of these factors enhances the risk of the emergence of antibiotic resistance [10] and this risk could be compounded by resistant strain circulation. To reflect these conditions accurately, we have chosen Swaziland (also now called the Kingdom of Eswatini; a nation in southern Africa) as a model population. With 27.4% of its adult population being HIV/AIDS-positive (HIV/AIDS+) in 2015 [15] (down from 32% among adults aged 18-49 in 2013; [16]), Swaziland represents a worst-case scenario of host vulnerability to drug-resistant opportunistic infection.

To explore the evolutionary and epidemiological effects of the emergence and subsequent circulation of antibiotic-resistant pathogens within a highly immunocompromised host population, we present an SEIR model [17], and parameterize it to reflect conditions similar to those in Swaziland (as in [8,10]). We vary the percentage of HIV/AIDS+ susceptibles using antibiotic prophylaxis (thereby protecting themselves from drug-sensitive pathogens), and the probability of resistant strain transmission, including the potential for either increased or decreased fitness of the resistant strain, relative to that of the wild-type. We define successful strain fitness as successful transmission, whether due to altered rates of within-host replication yielding altered exposure per contact, or altered probability of successful transmission from the same level of bacterial exposure. By analyzing the condition-dependent differences in the evolutionary success of drug-sensitive and drug-resistant bacterial strains, we provide a framework for developing public health policy recommendations geared toward minimizing the emergence and proliferation of resistance.

## 2. Results

**Figure 1:**
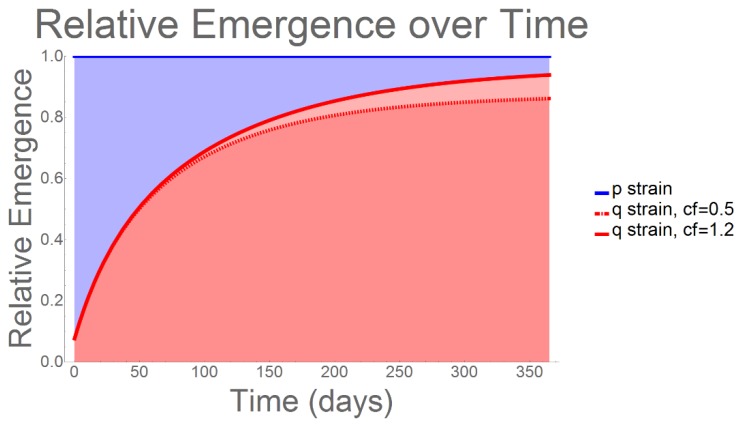
Relative Emergence: Using the extremal comparators of resistant strain comparative fitness (cf) = 0.5 and 1.2, we analyzed the resulting changes to relative emergence. Even when its evolutionary fitness is low (0.5), q-strain dominance occurs immediately and rapidly; among all infections, 80–95% can be expected to be antibiotic-resistant, irrespective of percent prophylaxis treatment among HIV/AIDS patients.

When we analyzed the evolutionary behavior of the resistant (q) strain over a one-year period, we observed an immediate and rapid increase emergence, such that, by day 365, more than 90% of infections could be expected to be attributable to q-strain emergence (Figure 1). This result occurred irrespective of the percentage of HIV/AIDS+ susceptibles being prescribed antibiotic prophylaxis. However, we note that, as the comparative fitness of the q-strain (again, relative to that of the p-strain) increased from 0.5 to 1.2 [18], we observed a corresponding increase in the percentage of infections associated with q-strain emergence.

**Figure 2:**
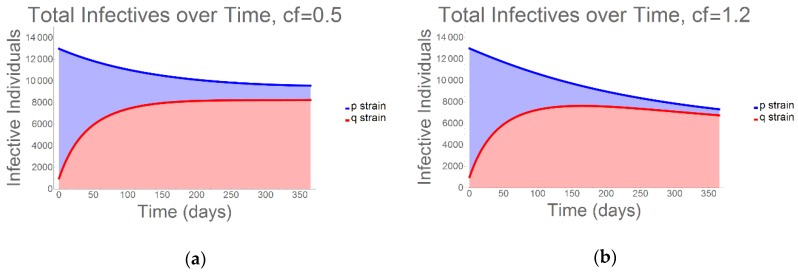
Total Infectivity: Again using (**a**) the lowest (cf = 0.5) and (**b**) the highest (cf = 1.2) resistant strain comparative fitness figures, we quantified the prevalence of the q-strain versus that of the p-strain for a one-year period. While there is a slight fitness-dependent change in the ratio of drug-sensitive to drug-resistant infections, the rapid q-strain dominance occurs even when its comparative fitness is low. Therefore, surveillance efforts that analyze total tuberculosis (TB) prevalence only, while failing to consider the percentage of resistant strain infectives within the population, could be critically flawed—especially when HIV/AIDS prevalence is high [10].

As would be expected, due to both curative antibiotics and a decline in available hosts (whether due to immune memory or mortality), we observed a decline in total infection prevalence over the 365-day duration of the model (Figure 2). Nevertheless, it is crucial to recognize the speed with which the resistant strain emerges and begins to outcompete the sensitive strain. Again, using the endpoint comparative fitness values of 0.5 and 1.2 for the resistant strain, the infection curves depicted in Figure 2 demonstrate that, while nearly identical with regard to population-wide tuberculosis (TB) prevalence, over just a short time resistant strain cases account for the majority of all infectives.

**Figure 3:**
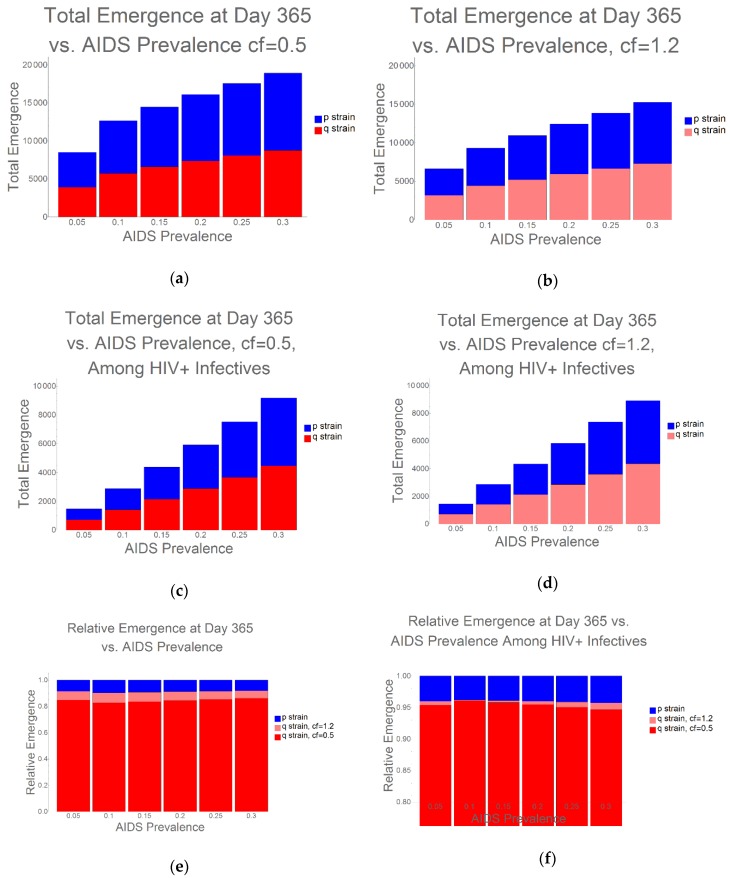
Ratios of Emergence as HIV/AIDS Prevalence Increases: We investigated the emergence and dominance of resistant strains as HIV/AIDS prevalence increased from 0%–30%, and we present results for the entire population, and for HIV/AIDS+ hosts only. Irrespective of the initial fitness of the q-strain, we observe increasing q-strain success as HIV/AIDS prevalence increases. (**a**,**b**) Emergence in the total population with (**a**) cf = 0.5 and (**b**) cf = 1.2; (**c**,**d**) Emergence in the HIV+ infective population with (**c**) cf = 0.5 and (**d**) cf = 1.2; (**e**,**f**) Relative emergence among (**e**) total population and (**f**) HIV+ infectives.

Even under the condition when the q-strain experiences the greatest fitness penalty (cf = 0.5), examining total TB prevalence, without analyzing the percentage of drug-resistant vs. drug-sensitive strains, would critically fail to capture overall risk: bacterial strains unresponsive to antibiotics have the potential to be particularly harmful to highly HIV/AIDS-immunocompromised populations. As further evidence of the health risk that arises due to the interplay between HIV/AIDS and emerging resistance, we found that, as HIV/AIDS prevalence increases, there is a corresponding increase in the proportion of q-strain infections, and this occurs irrespective of the relative fitness of the resistant strain (Figure 3).

**Figure 4:**
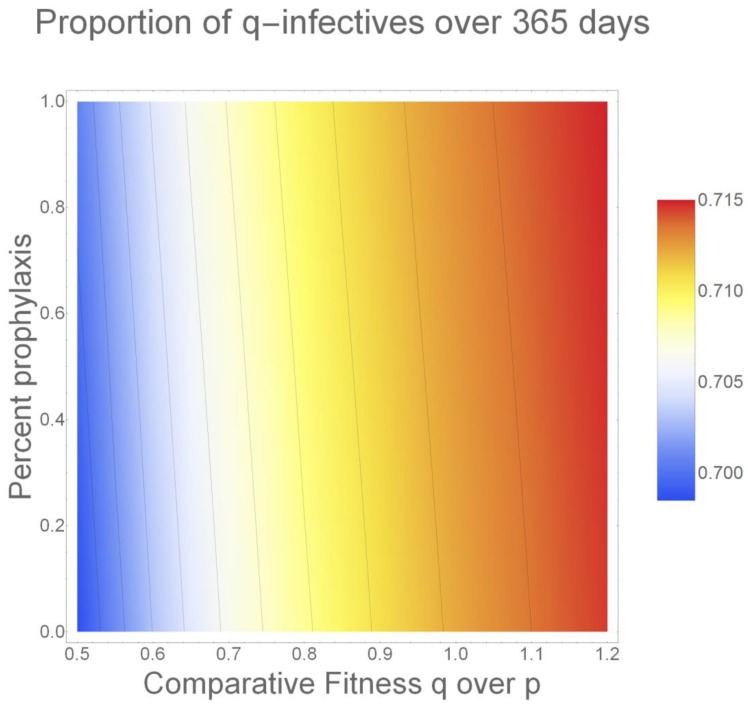
Combined Impact of Comparative Fitness and Percent Prophylaxis Use: Here, we present a heat map representing the change in q-strain prevalence that occurs due to prophylaxis use and changes in comparative fitness. We demonstrate that increased prophylaxis use, and increased comparative fitness, both benefit the resistant strain. While the magnitude of the effect of increased relative fitness is observably greater, even when the relative fitness of q is low, the selective pressure imposed by prophylaxis use increases q-strain prevalence overall (we note that the lines appearing on the heat map are present for visual assistance only).

Finally, Figure 4 illustrates the combined impact of percent prophylaxis use and resistant strain fitness. Visualizing these effects using a heat map allows us to analyze the importance of both selective pressures at once. Even when q-strain fitness is at its presumed lowest (cf = 0.5) [18], the selective pressure applied by HIV/AIDS patients’ use of prophylaxis increases q-strain emergence. While this effect is not as pronounced as the q-strain prevalence that we observe as its comparative fitness is increased, it is worth noting with respect to prophylaxis prescribing policies.

## 3. Discussion

Both the emergence of antibiotic resistance and the vulnerability of the HIV/AIDS-immunocompromised to opportunistic pathogens are well-documented medical crises [7,19,20,21,22,23,24,25]. In modeling the interplay between these two health risks, we recently demonstrated that the disrupted selective pressures associated with an HIV/AIDS-related host immunosuppression create the potential for a drastic, AIDS-attributable increase in the novel emergence of drug resistance [10].

In our current SEIR model, we examine the evolutionary impact of resistant strain emergence and circulation within a highly HIV/AIDS-prevalent host population. The results of this model highlight an additional reason why analyses of the probability of emergence of antibiotic resistance should include the consideration of population-wide HIV/AIDS prevalence: widespread use of medically recommended antibiotic prophylaxis is a phenomenon specific to highly immunocompromised host populations [26]. Therefore, the prophylaxis-attributable emergence of resistant microbial strains, as well as their subsequent circulation, is also directly related to population-level immunoincompetence. Our model demonstrates that, while the total number of infectives varied only slightly as prophylaxis use increased, the percentage of hosts infected within drug-resistant TB strains increased rapidly, thereby increasing the relative fitness of resistant TB strains.

The use of antibiotic prophylaxis is known to create an evolutionary tradeoff, wherein despite the potential improvement in host health, a fitness benefit is conferred to resistant bacterial strains [27]. However, within the context of an HIV/AIDS-prevalent host pool, the elevation of resistant strain fitness that arises as an inevitable by-product of prophylaxis use may represent a considerably greater health risk—especially given the speed with which resistant bacterial strains become dominant. By the end of the 365-day duration of our model, we find that more than 90% of TB infections could be expected to be antibiotic-resistant, even when resistant strain fitness is comparatively low. While this percentage may seem high, Sanchez-Padilla, et al. [28], found that, in Swaziland, more than 50% of culture-positive TB patients harbored resistant strains as of 2010.

In a populations such as Swaziland, in which up to 27.4% of the adult population may be immunocompromised [15], this means that a large proportion of the host pool could become infected with bacterial strains that exhibit little, if any, response to targeted antibiotics. In the absence of both antibiotic treatment and sufficient immune response to fight infection, elevated morbidity and mortality among HIV/AIDS+ hosts are likely outcomes (though to a lesser degree, it is also possible that fully immunocompetent hosts will suffer the effects of resistant strain dominance, as emergence initially arising in response to the selective pressure imposed by antibiotic prophylaxis is maintained via host mixing and strain circulation).

We note that, without violating our simplifying assumption that immune status remains unchanged over the duration of the model, we are limited in our ability to assess the long-term (>one year) behavior of this system. However, our initial findings suggest that surveillance efforts directed toward examining the prevalence of TB—or any other opportunistic pathogen—alone, without consideration for strain specificity, will fail to capture the impact of HIV/AIDS-related effects, such as the widespread use of antibiotic prophylaxis, on resistant strain emergence and maintenance. Moreover, it is likely that projections regarding TB-attributable morbidity and mortality will be underestimated if the percentage of hosts infected with resistant bacterial strains is not taken into account. Given that these effects were visible within the one-year duration of this model, the potential for long-term resistant-strain dominance creates a public health threat that cannot be ignored.

## 4. Materials and Methods 

### 4.1. Mathematical Model

Extending our previous work, we stratify our population based on immune status, including five categories of susceptibles: (1) those who are fully immunocompetent (i.e., HIV/AIDS-negative); (2) those who are HIV+ or AIDS+, but whose opportunistic infection risk is minimized by consistent use of HAART [29,30,31]; (3) those whose are AIDS-immunocompromised (i.e., HAART-). We further divide the susceptible HIV/AIDS+, HAART+, and AIDS+, HAART- subpopulations into those who as an initial condition, who receive antibiotic prophylaxis, and those who do not [8,10]. We note that we have made the simplifying assumption that no seroconversion occurs during the duration of the model. We recognize that making this assumption limits the timeframe for our analysis. We therefore examine the evolutionary fitness of the resistant and wild-type strains during a one-year period, within which we may observe the emergence of antibiotic resistance [32] and assess the initial behavior of the system, without having to account for changes in host immune status [29]. Future work will explore the longer-term implications of bacterial strain competition under shifting conditions of HIV/AIDS.

Within each immune class, we further delineate our population based on the combination of bacterial infection and adherence to targeted antibiotics. The combined description of immune/HAART and infection/antibiotic status is depicted via super- and subscripts to the variables associated with each compartment of the model. ^Superscripts^ dually describe immune and HAART status, and _subscripts_ are used to dually describe bacterial infection status and antibiotic adherence, such that _- -_ means infection negative (and, therefore, untreated); _* +_ means infection-positive, completely adherent; _* /_ means infection positive, nonadherent; and _* -_ means infection-positive, untreated. For purposes of this description, we use “*” generically to represent the possibility of infection with either the drug-sensitive or drug-resistant bacterial strain. In the ODE model equations (Appendix A), however, we use notation reflecting Hardy–Weinberg models [33], in which “p” represents the wild-type allele, and “q” represents the mutant allele, to distinguish between the drug-sensitive (wild-type) and drug-resistant (mutant) strains among actively infected hosts (for example, whereas _p +_ indicates that the host is infected with a wild-type strain, _q +_ indicates that the host is infected with a mutant strain; in both cases the “+” designates complete antibiotic adherence). Finally, we use the subscript _- +_ to describe prophylactically treated susceptibles.

For HIV/AIDS+ susceptibles initially prescribed antibiotic prophylaxis, we make two conservative assumptions: First, we assume that these susceptibles are completely adherent to their prophylaxis regimens. Second, we assume that those who contract drug-resistant infections while prophylactically treated are then also completely adherent to the targeted antibiotics subsequently prescribed to treat the infection. Therefore, all infectives who have previously been treated prophylactically are assigned to either the $I_{q+}^{+-}$ or $I_{q+}^{++}$ categories, depending on HIV/HAART status.

Particularly in the developing world, many demographic and economic factors influence host antibiotic adherence [34,35,36,37,38,39]. Therefore, using the constant “C,” we divide infectives based on the probability that they will participate in each of three categories as follows: C_+_ is the probability of immediate infection detection and complete treatment, as defined by DOTS protocol [40]; C_/_ is the probability of partial adherence, wherein the host received antibiotics for some period of time during infectivity, but did not follow dosing instructions; and C_-_ is the probability that the host failed to seek treatment.

$\beta_{q}^{n}$ (where “n” is used to represent any possible immune status) values are derived from the work of Cohen and Murray [41], who provide a transmission rate constant of 8.5 × 10^−6^ for drug-resistant TB among immunocompetent hosts. We adjusted Cohen and Murray’s rate, which was based on an idealized population of one million, to account for the combination of Swaziland’s total population, and the size of each immune status-based subpopulation, and converted it from an annual to a daily rate. In assigning β values associated with both the sensitive and resistant strains, we assume equivalent immune function for the fully immunocompetent and HAART+ categories. We also assume that the actively AIDS-immunocompromised are an arbitrary 10% more likely to contract TB following exposure to an infected individual.

Since data regarding the transmission probability of drug-sensitive TB were not immediately available in the literature, we assigned values to $\beta_{p}^{n}$ using the combination of the comparative fitness results set forth by Cohen, et al. [18], and the drug-resistant TB transmission probability published by Cohen and Murray [41]. Details regarding the assignment of parameter values, including those for $\beta_{p}^{n}$, appear in [8,10] and are further described in Appendix A.

We use ϕ to represent the composite probability of the emergence and success of an antibiotic-resistant infection among prophylactically treated susceptibles. Values for ϕ were determined based on the per cell, per bacterial generation mutation rate; the total number of infected cells per host; the expected number of bacterial generations per infection duration; the per category infection duration; and the relative success of the mutant strain [42,43,44,45]. We note that ϕ is used to represent the probability of resistance arising out of mutation only and does not represent the probability of contracting a resistant infection due to strain circulation.

Finally, we use ζ to represent the immune status-based transition rate from latent to active infection. Adapting our values from Cohen and Murray [41], who themselves rely upon Dye [46,47], we chose an annualized, midrange transition rate of 0.88, and, consistent with the other parameters used in this model, and adjusted it to reflect a daily transition probability. We assume that those with active AIDS progress from exposed to infective 10% faster than those who are HIV/AIDS- or HAART+, and that those who are HAART+ progress at the same rate as the fully immunocompetent. We also assume that ζ values are equivalent for the drug-sensitive and drug-resistant TB strains.

**Figure 5:**
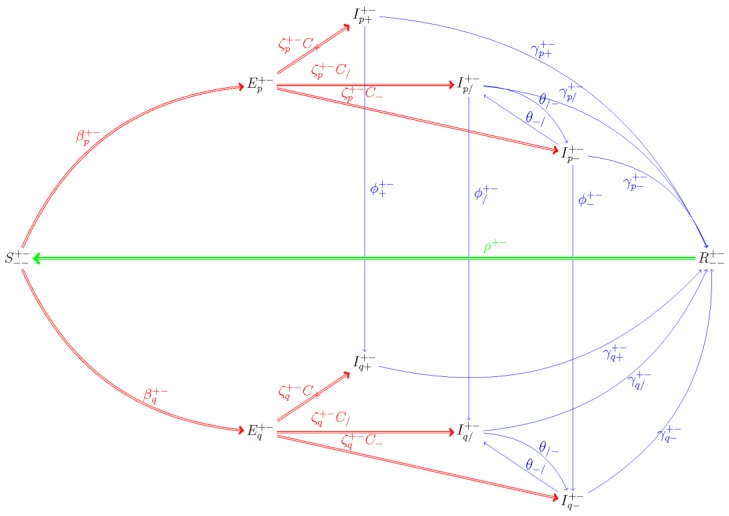
Compartmental Susceptible-Exposed-Infectious-Recovered (SEIR) Model: The model follows the progression of fully immunocompetent, HIV/AIDS+, HAART- and HIV+/HAART+ susceptibles who become infected with either drug-sensitive (p) or drug-resistant (q) TB strains. As an example, we include a diagram depicting this process for actively AIDS-immunocompromised ( $S_{--}^{+-}$ ) susceptibles who have not previously received antibiotic prophylaxis.

Our model follows the progression of both drug-sensitive and drug-resistant infections throughout a population stratified based on immune status (Figure 5). We use a set of ordinary differential equations to describe the system (Appendix A), where the symbols $I_{all,p}$ and $I_{all,q}$ are used to represent the sum of all of those infected with drug-sensitive or drug-resistant infections, respectively, that can that infect susceptibles at a rate of β; β depending on both immune status, and strain type. We include three separate mortality rates: $\omega_{I}$ represents rate of death due to TB; $\omega_{A}$ represents AIDS-attributable rate of death; and ω represents all other cause-related rate of death. Other parameters used include α, which represents per capita birthrate; ρ, which represents the immune-status-dependent rate of loss of immunity; γ, which represents the HIV/AIDS and antibiotic-category-dependent rate of recovery from bacterial infection; θ, which represents the rate of transition between the partially antibiotic adherent and untreated states; and ψ, which represents the HAART-dependent increase in infection-attributable death for patients with active AIDS (a detailed list of parameters, including their condition dependencies, values used, and the reference(s) from which they were estimated, where applicable, and the methods by which other parameter values were calculated are all detailed in [8,10]).

Model outcomes were obtained by implementation of the model in the Wolfram Mathematica programming language (see Supplemental File S1).

### 4.2. Methodological Framework

Using HIV/AIDS prevalence data from Swaziland, we address the question of evolutionary fitness in drug-resistant and drug-sensitive bacterial strains when prophylaxis use among the HIV/AIDS-immunocompromised and resistant strain transmission probability are varied (we include a parameter corresponding to the probability of curative antibiotic adherence among the actively infective, but we assume that prophylactically-treated HIV/AIDS+ susceptibles who become infective cease treatment with broad-spectrum prophylaxis during the infectious period).

### 4.3. Prophylaxis-Attributable Emergence and Strain Circulation

The use of antibiotic prophylaxis has been heavily relied upon as a means of opportunistic infection management among the HIV/AIDS-immunocompromised [20]. However, despite its protective value to HIV/AIDS patients, use of broad-spectrum prophylaxis can select for the emergence of resistance [48]. With the expectation that the prevalence of prophylaxis use in the developing world may vary significantly based on factors such as drug availability, patient age and/or socioeconomic status and the drug’s side-effect profile [49,50,51], we capture the combined impact of primary, prophylaxis-attributable emergence, and the secondary infections that occur due to emergent strain circulation, as percent prophylaxis treatment among HIV/AIDS+ susceptibles is increased incrementally from 0% to 100%.

### 4.4. Host-Dependent Variation in Transmission Probability

Pathogen persistence relies upon the composite probability of host-to-host contact and infection transmission [52]. Host susceptibility increases the likelihood of pathogen success; with greater susceptibility—as would be the case in an AIDS-prevalent host population—comes the potential for more widespread transmission [53]. Among immunocompromised hosts, however, transmission potential is mitigated by the increased likelihood of host mortality—especially in the absence of antibiotic treatment that can occur due to host nonadherence and/or drug resistance.

Cohen, et al. [18] found that the comparative fitness values of certain drug-resistant TB mutants ranged from 0.5 to 1.2, relative to their parent strains. We used this same range to analyzed fitness-based differences in the prevalence of drug-sensitive and drug-resistant TB over a one-year period.

## Supplementary Materials

The following are available online at https://www.mdpi.com/2079-6382/9/3/114/s1, Supplementary File S1: Computer Code.

## Appendix A. Model Description, Equations, and Parameters

### Appendix A.1. Model Description and Parameters

In this paper, we build on the existing models in previous work [8,10] to account for pathogen co-circulation within the host pool. The immune and antibiotic treatment statuses of those individuals corresponding to the “susceptible” compartment are described using the same convention (e.g., HIV/AIDS- susceptibles are represented as $S_{--}^{--}$) as in [8,10]. However, exposed, infective and recovered members of the population are now categorized by pathogen strain type. Borrowing descriptors from the Hardy–Weinberg model [33], we use “p” in the subscript to describe wild-type (drug-sensitive) strains, and “q” to describe mutant (drug-resistant) strains. The second portion of the subscript remains identical to the system used previously. For example, an HIV/AIDS+, HAART- individual who contracts a drug-resistant infection and is fully antibiotic-adherent is represented as $I_{q+}^{+-}$ (we note that, among those in the exposed category, only the first part of the subscript descriptor is used, since antibiotic adherence status only applies to those who are actively infective).

The model, composed of the system of ordinary differential equations below, follows the progression of TB infection through a population stratified by immune and antibiotic status. Parameter values and the sources thereof are described in [8,10]; however, note that we now include ζ values corresponding to the transition rate from to the actively infective state once exposed to an antibiotic-resistant infection. We assume that ζ values corresponding to the mutant strain are equivalent to those corresponding to the wild-type strain (previously discussed) and all values are immune status-dependent.

### Appendix A.2. System of Differential Equations

The model is described by the following system of 38 differential (plus two algebraic) equations.

(A1) dS−−−−dt=−βp−−S−−−−Iall,p−βq−−S−−−−Iall,q+ρ−−R−−−−+α(S−−−−+Ep−−+Eq−−+Ip+−−+R−−−−+S−−++         +Ep+++Eq+++Ip++++R−−+++S−++++Eq2+++R−+++)−ωS−−−−

(A2) dS−−+−dt=−βp+−S−−+−Iall,p−βq+−S−−+−Iall,q+ρ+−R−−+−+α(S−−+−+Ep+−+Eq+−+Ip++−+R−−+−+S−++−+Eq2+−+R−−+−)−(ω+ωv)S−−+−

(A3) dS−++−dt=−βq+−S−++−Iall,q+ρ+−R−++−+α(S−++−+Eq2+−+Iq2+−+R−++−)−(ω+ωv)S−++−

(A4) dS−−++dt=−βp++S−−++Iall,p−βq++S−−++Iall,q+ρ++R−−++−ωS−−++

(A5) dS−+++dt=−βq++S−+++Iall,q+ρ++R−+++−ωS−+++

(A6) $$I_{all,p}=I_{p+}^{--}+I_{p/}^{--}+I_{p-}^{--}+I_{p+}^{+-}+I_{p/}^{+-}+I_{p-}^{+-}+I_{p+}^{++}+I_{p/}^{++}+I_{p-}^{++}$$

(A7) $$I_{all,q}=I_{q+}^{--}+I_{q/}^{--}+I_{q-}^{--}+I_{q+}^{+-}+I_{q/}^{+-}+I_{q-}^{+-}+I_{q+}^{++}+I_{q/}^{++}+I_{q-}^{++}+I_{q2}^{+-}+I_{q2}^{++}$$

(A8) dEp−−dt=βp−−S−−−−Iall,p−ζp−−Ep−−−ωEp−−

(A9) dEq−−dt=βq−−S−−−−Iall,q−ζq−−Eq−−−ωEq−−

(A10) dEp+−dt=βp+−S−−+−Iall,p−ζp+−Ep+−−(ω−ωv)Ep+−

(A11) dEq+−dt=βq+−S−−+−Iall,q−ζq+−Eq+−−(ω−ωv)Eq+−

(A12) dEp++dt=βp++S−−++Iall,p−ζp++Ep++−ωEp++

(A13) dEq++dt=βq++S−−++Iall,q−ζq++Eq++−ωEq++

(A14) dEq2+−dt=βq+−S−++−Iall,q−ζq+−Eq2+−−(ω−ωv)Eq2+−

(A15) dEq2++dt=βq++S−+++Iall,q−ζq++Eq2++−ωEq2++

(A16) dIp+−−dt=C+ζp−−Ep+−−ϕ+−−Ip+−−−γp+−−Ip+−−−ωIp+−−

(A17) dIq+−−dt=C+ζq−−Eq+−+ϕ+−−Ip+−−−γq+−−Iq+−−−ωIq+−−

(A18) dIp/−−dt=C/ζp−−Ep−−−ϕ/−−Ip/−−−θ/−Ip/−−+θ−/Ip−−−−γp/−−Ip/−−+α(Ip/−−+Ip/++)−(ω+ωb)Ip/−−

(A19) dIq/−−dt=C/ζq−−Eq−−+ϕ/−−Ip/−−−θ/−Iq/−−+θ−/Iq−−−−γq/−−Iq/−−+α(Iq/−−+Iq/++)−(ω+ωb)Iq/−−

(A20) dIp−−−dt=C−ζp−−Ep−−−ϕ−−−Ip−−−+θ/−Ip/−−−θ−/Ip−−−−γp−−−Ip−−−+α(Ip−−−+Ip−++)−(ω+ωb)Ip−−−

(A21) dIq−−−dt=C−ζq−−Eq−−+ϕ−−−Ip−−−+θ/−Iq/−−−θ−/Iq−−−−γq−−−Iq−−−  +α(Iq−−−+Iq−+++Iq+−−+Iq++++Iq2++)−(ω+ωb)Iq−−−

(A22) dIp++−dt=C+ζp+−Ep+−−ϕ++−Ip++−−γp++−Ip++−−(ω+ωv)Ip++−

(A23) dIq++−dt=C+ζq+−Eq+−+ϕ++−Ip++−−γq++−Iq++−+αIq++−−(ω+ωv)Iq++−

(A24) dIp/+−dt=C/ζp+−Ep+−−ϕ/+−Ip/+−−θ/−Ip/+−+θ−/Ip−+−−γp/+−Ip/+−+αIp/+−−(ω+ψωb+ωv)Ip/+−

(A25) dIq/+−dt=C/ζq+−Eq+−+ϕ/+−Ip/+−−θ/−Iq/+−+θ−/Iq−+−−γq/+−Iq/+−+αIq/+−−(ω+ψωb+ωv)Iq/+−

(A26) dIp−+−dt=C−ζp+−Ep+−−ϕ−+−Ip−+−+θ/−Ip/+−−θ−/Ip−+−−γp−+−Ip−+−+αIp−+−−(ω+ψωb+ωv)Ip−+−

(A27) dIq−+−dt=C−ζq+−Eq+−+ϕ−+−Ip−+−+θ/−Iq/+−−θ−/Iq−+−−γq−+−Iq−+−+αIq−+−−(ω+ψωb+ωv)Iq−+−

(A28) dIp+++dt=C+ζp++Ep++−ϕ+++Ip+++−γp+++Ip+++−ωIp+++

(A29) dIq+++dt=C+ζq++Eq+++ϕ+++Ip+++−γq+++Iq+++−ωIq+++

(A30) dIp/++dt=C/ζp++Ep++−ϕ/++Ip/++−θ/−Ip/+++θ−/Ip−++−γp/++Ip/++−(ω+ωb)Ip/++

(A31) dIq/++dt=C/ζq++Eq+++ϕ/++Ip/++−θ/−Iq/+++θ−/Iq−++−γq/++Iq/++−(ω+ωb)Iq/++

(A32) dIp−++dt=C−ζp++Ep++−ϕ−++Ip−+++θ/−Ip/++−θ−/Ip−++−γp−++Ip−++−(ω+ωb)Ip−++

(A33) dIq−++dt=C−ζq++Eq+++ϕ−++Ip−+++θ/−Iq/++−θ−/Iq−++−γq−++Iq−++−(ω+ωb)Iq−++

(A34) dIq+−dt=ζq+−Eq2+−−γq2+−Iq2+−+αIq2+−−(ω+ωv)Iq2+−

(A35) dIq2++dt=ζq++Eq2++−γq2++Iq2++−ωIq2++

(A36) dR−−−−dt=γp+−−Ip+−−+γp/−−Ip/−−+γp−−−Ip−−−+γq+−−Iq+−−+γq/−−Iq/−−+γq−−−Iq−−−−ρ−−R−−−−−ωR−−−−

(A37) dR−−+−dt=γp++−Ip++−+γp/+−Ip/+−+γp−+−Ip−+−+γq++−Iq++−+γq/+−Iq/+−+γq−+−Iq−+−−ρ+−R−−+−−(ω+ωv)R−−+−

(A38) dR−−++dt=γp+++Ip++++γp/++Ip/+++γp−++Ip−+++γq+++Iq++++γq/++Iq/+++γq−++Iq−++−ρ++R−−++−ωR−−++

(A39) dR−++−dt=γq2+−Iq2+−−ρ+−R−++−−(ω+ωv)R−++−

(A40) dR−+++dt=γq2++Iq2++−ρ++R−+++−ωR−+++
